# Inflammatory cytokine-induced changes in neural network activity measured by waveform analysis of high-content calcium imaging in murine cortical neurons

**DOI:** 10.1038/s41598-017-09182-5

**Published:** 2017-08-22

**Authors:** Benjamin D. S. Clarkson, Robert J. Kahoud, Christina B. McCarthy, Charles L. Howe

**Affiliations:** 1 Department of Neurology, Mayo Clinic, Rochester, MN USA 55905 USA; 2 Department of Pediatrics, Mayo Clinic, Rochester, MN USA 55905 USA; 3 Department of Neuroscience, Mayo Clinic, Rochester, MN USA 55905 USA; 4 Department of Immunology, Mayo Clinic, Rochester, MN USA 55905 USA; 5 Center for Multiple Sclerosis and Autoimmune Neurology, Mayo Clinic, Rochester, MN USA 55905 USA

## Abstract

During acute neuroinflammation, increased levels of cytokines within the brain may contribute to synaptic reorganization that results in long-term changes in network hyperexcitability. Indeed, inflammatory cytokines are implicated in synaptic dysfunction in epilepsy and in an array of degenerative and autoimmune diseases of the central nervous system. Current tools for studying the impact of inflammatory factors on neural networks are either insufficiently fast and sensitive or require complicated and costly experimental rigs. Calcium imaging offers a reasonable surrogate for direct measurement of neuronal network activity, but traditional imaging paradigms are confounded by cellular heterogeneity and cannot readily distinguish between glial and neuronal calcium transients. While the establishment of pure neuron cultures is possible, the removal of glial cells ignores physiologically relevant cell-cell interactions that may be critical for circuit level disruptions induced by inflammatory factors. To overcome these issues, we provide techniques and algorithms for image processing and waveform feature extraction using automated analysis of spontaneous and evoked calcium transients in primary murine cortical neuron cultures transduced with an adeno-associated viral vector driving the GCaMP6f reporter behind a synapsin promoter. Using this system, we provide evidence of network perturbations induced by the inflammatory cytokines TNFα, IL1β, and IFNγ.

## Introduction

Immune mediators and inflammatory cytokines have been implicated in synaptic dysfunction in an array of CNS inflammatory and autoimmune diseases, including multiple sclerosis^1–3^, autoimmune epilepsy, limbic encephalopathy^4^, febrile infection-related epilepsy syndrome^5, 6^, and post-traumatic epilepsy^7, 8^. A growing body of evidence also suggests that inflammatory factors may facilitate ictogenesis and epileptogenesis in many patients with “traditional” epilepsy^9–12^. However, the field is generally lacking fast, sensitive, and readily implemented tools for measuring the impact of inflammatory factors on neural networks and for screening potential immunomodulatory drugs for the capacity to restore neuronal activity patterns.

Current strategies for medium-to-high throughput screening of potentially neuroactive compounds largely rely on multi-well microelectrode arrays. Such arrays use non-invasive detection of extracellular field potentials in dissociated neuronal cultures to quantify spike frequencies and voltage waveforms and to measure neuronal synchronicity based on cross-correlation of spike events^13–18^. However, given the current size and spatial resolution of commercially available arrays the extracellular field potentials recorded by microelectrodes represent aggregate activity patterns arising from numerous neurons adjacent to the electrode while leaving many neurons in the network unobserved^19^. Recent advances using complementary metal-oxide semiconductor-based multi-electrode arrays have increased the spatial resolution of these approaches^20–25^, but these platforms have tradeoffs in total recording area and signal-to-noise ratio. In contrast, live cell imaging of action potential-associated calcium transients in primary neuron cultures provides single cell-resolution of activity patterns while still achieving simultaneous recording in hundreds of neurons^19^.

An important consideration in designing a culture platform for screening inflammatory mediators or immunomodulatory drugs is that these factors may indirectly impact neuronal function via effects on glial cells^26^. Thus, while it is necessary to have glia in the culture system in order to capture physiologically relevant cell-cell interactions, such heterogeneity may confound automated analyses that cannot distinguish between glial and neuronal calcium transients. Recent advances in genetically encoded calcium indicators (GECIs) provide a means for stable cell-specific expression of calcium indicators with high signal-to-noise ratios and rapid fluorescence kinetics^27^. These indicators also allow repeated measures of calcium in neurons over substantially longer time frames than conventional fluorescent dye indicators that must be loaded into cells at the time of imaging and which have poor toxicity profiles. Longitudinal imaging using stably expressed GECIs facilitates the analysis of activity pattern evolution in neurons as they mature and permits comparisons between baseline and post-treatment measurements of neuronal activity.

Herein, we provide techniques and algorithms for image processing and waveform feature extraction using automated analysis of calcium transients in primary murine cortical neuron cultures infected with an adeno-associated viral vector driving the GCaMP6f reporter behind a synapsin promoter. In addition to analysis of activity evolution in the neurons through time *in vitro*, we provide evidence of network perturbations induced by the inflammatory cytokines tumor necrosis factor alpha (TNFα), interleukin-1 beta (IL1β), and interferon gamma (IFNγ).

## Results

### Characterization of calcium transients in high-density cortical neuron cultures expressing GCaMP6f

**Figure 1:**
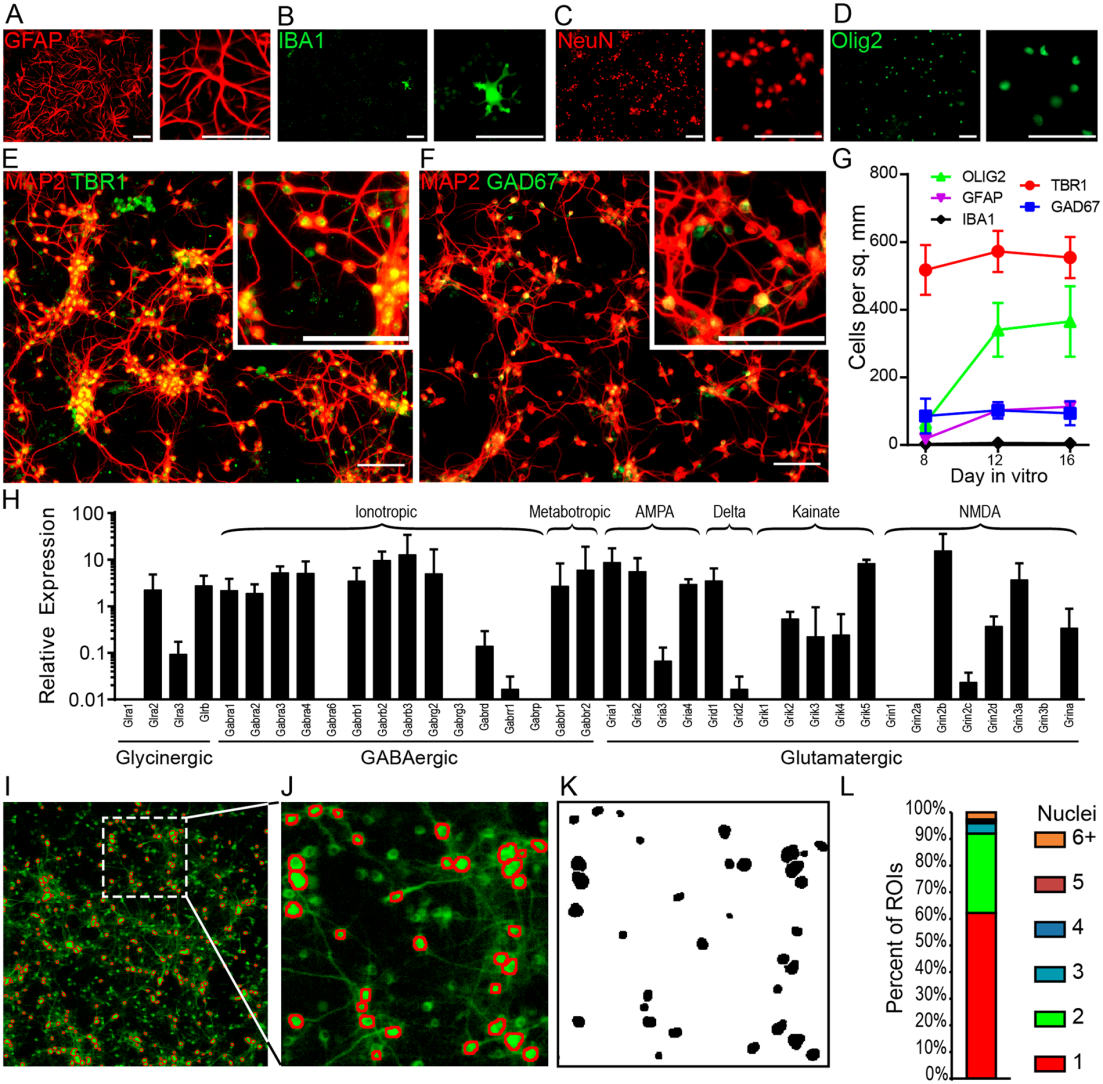
Automated GCaMP6f fluorescent image segregation for tracking neuronal calcium transients in high-density cultures. Representative immunostaining for (**A**) glial fibrillary acid protein (GFAP; astroglia), (**B**) ionized calcium binding adaptor molecule 1 (Iba1; microglia), (**C**) neuronal nuclei (NeuN; neurons), (**D**) oligodendrocyte lineage transcription factor 2 (Olig2; oligodendrocytes), (**E**,**F**) microtubule associated protein 2 (MAP2; neurons), (**E**) T-box brain 1 (TBR1; glutamatergic neurons), (**F**) glutamate decarboxylase molecular weight 67 kD (GAD67; GABA-ergic neurons). 10x fluorescent micrographs were acquired on DIV8, 12, and16 using an LSM 780 confocal microscope. Scale bar is 50 μm. (**G**) Quantification of immunostaining for cellular markers from DIV8 to DIV16. Positively labeled cells were manually counted in 4–10 optical fields. Error bars are mean +/− 95% confidence intervals (CI). (**H**) The relative expression of excitatory and inhibitory neurotransmitter receptors in the cultures was determined by RTPCR. (**I**–**L**) Neuronal cultures were infected at plating with 2000 MOI of AAV1.Syn.GCaMP6f.WPRE.SV40. At time of acquisition culture plates were transferred to the stage of a Zeiss 5 Live spinning disc confocal microscope with environmental chamber. After brief environmental equilibration, 250 frames were captured by time series acquisition at 5 frames per second. Maximum intensity projections were generated from the time series and imported into ImageJ for automated region of interest (ROI) generation. (**I**–**J**) Maximum intensity projection of raw time series acquisition with derived ROIs overlaid in red. (**K**) Binary image masks used for generating ROIs in (**J**,**L**) To determine correspondence between ROIs and individual cell bodies, cells were counterstained with DAPI immediately prior to imaging. ROIs were generated and overlaid on maximum intensity projections. The number of DAPI+ nuclei per ROI determined using ImageJ Cell Counter plugin for 494 ROIs. The percent of ROIs containing 1 or more nuclei is graphed. As shown, 90% of ROIs corresponded to 2 or fewer cell bodies. Findings are representative of two independent cultures.

Cortical neuronal progenitor cells derived from embryonic day 15 (E15) mice were seeded onto poly-ornithine coated tissue culture vessels at a density of 5 × 10^5^ cells per cm^2^. Immunofluorescent labeling of the cells after 8 days *in vitro* (DIV) revealed that a majority of these cells differentiated into MAP2^+^NeuN^+^ neurons (~90%; Fig. 1C). A substantial minority expressed markers of astrocytes (GFAP, ~2%; Fig. 1A) or oligodendrocytes (Olig2, ~8% Fig. 1D). A sparse population of IBA1^+^ microglial cells was also observed (<1%; Fig. 1B). Notably, the relative abundance of glial cells was substantially lower than what is observed *in vivo*. Of cells expressing neuronal markers, the majority were TBR1^+^ (86%; Fig. 1E), indicating glutamatergic specification^28^, while 14% of the neurons expressed glutamate decarboxylase (GAD67), a key enzyme for gamma-amino butyric acid (GABA) synthesis in GABA-ergic neurons (Fig. 1F). This ratio of glutamatergic to GABAergic neurons (~6:1) is similar to the tightly regulated 5:1 ratio reported for cortical neurons *in vivo* ^29^ and remained constant from day *in vitro* 8 through 16, despite increases in the percentage of astrocytes and oligodendrocytes (Fig. 1G). The transcriptional expression profile for glutamate, glycine, and GABA receptors in the culture system was determined by RT-PCR and indicates a broad pattern of excitatory and inhibitory synaptic connectivity (Fig. 1H).

In order to drive neuronal expression of GCaMP6f, cultures were infected at the time of seeding with 2000 viral particles per cell (6 × 10^9^ genome copies per 3 × 10^6^ cells) of AAV1.Syn.GCaMP6f.WPRE.SV40. Under these conditions, no increase in cellular toxicity was observed compared to uninfected controls (Figures S1). Expression of the vector is first detectable at DIV5, corresponding to the reported timing of synapsin expression in neural cultures derived from E15–E18 fetuses^30–32^. For analysis, fluorescence images were collected every 100 msec for 10 min and digitally stacked (Fig. 1I). A maximum intensity projection of the stack was then segregated into cellular regions of interest (ROI) using background normalization, thresholding, noise reduction, and size-limited particle analysis (Fig. 1J,K). Based on manual counting of nuclei contained within each ROI, this strategy predominantly isolated single neurons (65%) or regions containing two tightly apposed neuron cell bodies (31%); less than 10% of the ROIs contained three or more cells (Fig. 1L).

### Development of low-frequency, synchronous calcium transients in maturing high-density cortical neuron networks

**Figure 2:**
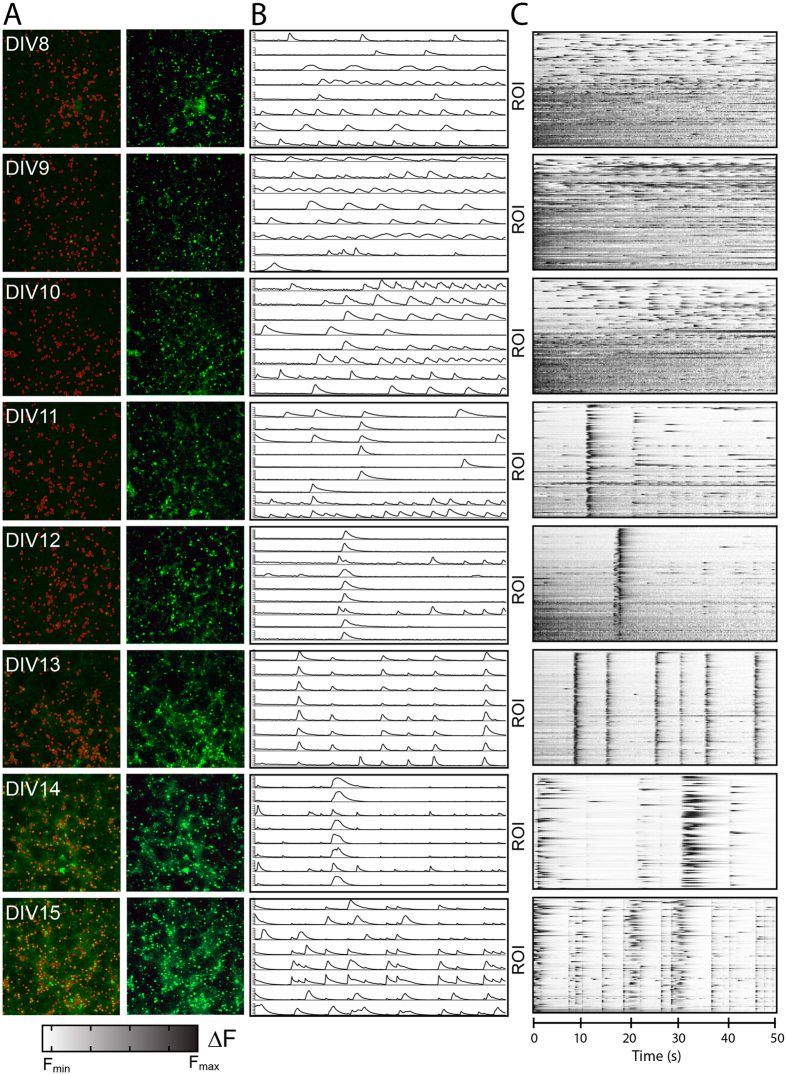
Automated multi-neuron tracking of calcium transients during maturation of neuronal cultures. (**A**) Representative maximum intensity projections of neurons at DIV8–DIV15 with (left) or without (right) ROI overlays (shown in red). Images on right are leveled. (**B**) Representative calcium traces for 8 individual neurons are shown for each time point. (**C**) Representative raster plots show Δ fluorescence traces in the 100 most active ROIs within the optical field for frames 1–250. Data are representative of 4 independent experiments and >50,000 total ROIs.

**Figure 3:**
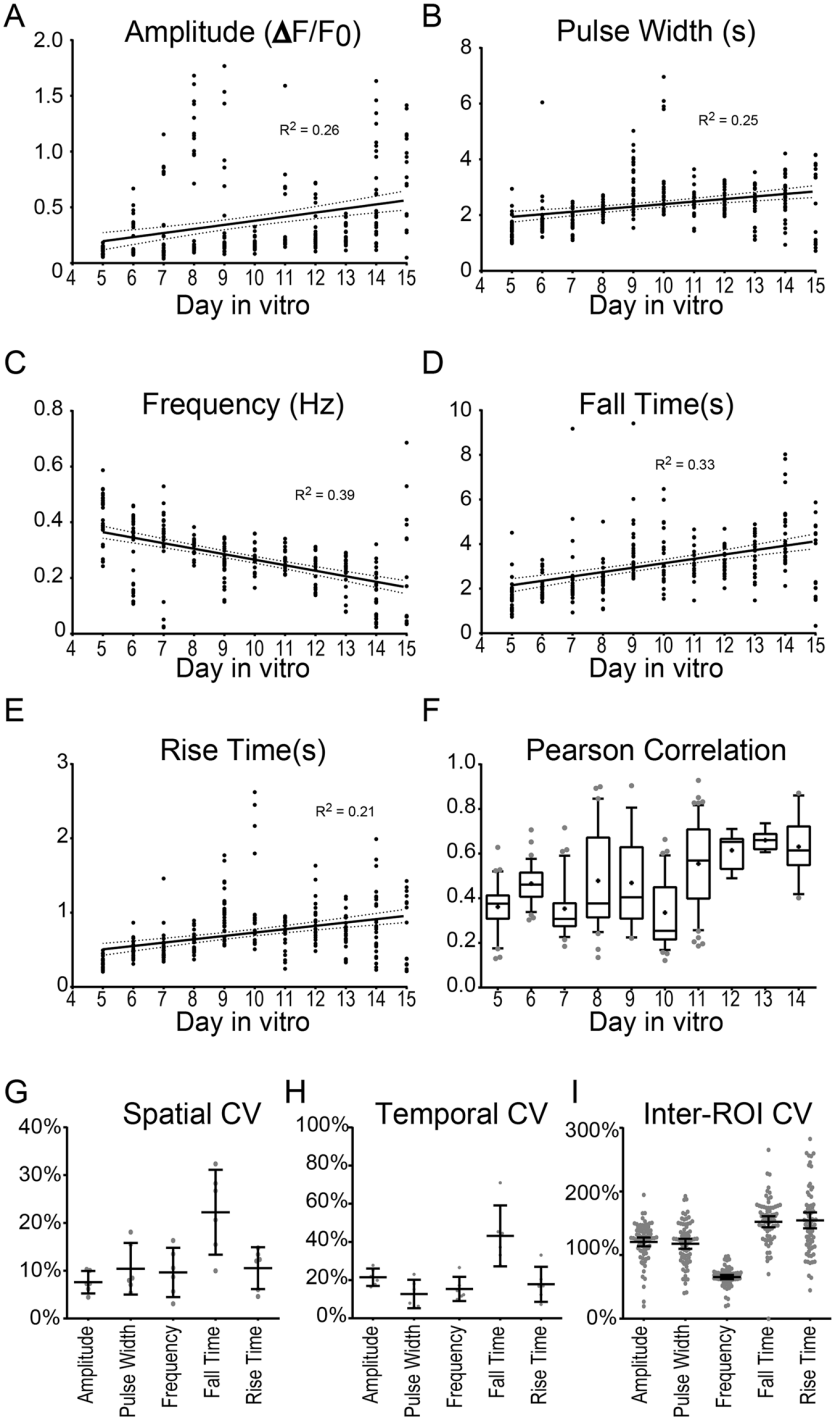
Waveform analysis of neuronal calcium transients. Raw Δ fluorescence traces from neuronal cultures (DIV8–DIV15) were analyzed using the Matlab Signal Processing Toolbox, to extract amplitude (**A**), pulse width (**B**), frequency (**C**), fall time (**D**), and rise time (**E**) for each ROI. (**F**) Synchronization was assessed by calculation of the Pearson correlation coefficient. Plots show averaged values from >16 acquisitions at each time point taken from 4 independent experiments. Solid lines represent linear regressions with 95% CI (dotted line). (**G**–**I**) Calcium traces were acquired as above from DIV14 cortical neuron cultures. (**G**) Spatial coefficient of variance (CV) for each of the computed waveform parameters was determined by comparing average values across multiple optical fields within the same well (n = 6 fields per well). (**H**) Temporal CV was determined for each waveform parameter by comparing the average values across serial acquisitions from the same optical field (n = 6 serial captures). (**I**) The coefficient of variation among all ROIs from a given optical field for each of the indicated metrics was calculated for n = 74 separate acquisitions (each dot represents one acquisition). Data are representative of 2 independent neural cultures.

Similar to reports based on hippocampal neurons^33^, we found that cortical neurons initially exhibit unsynchronized calcium oscillations. However, as the cells mature and form synaptic contacts, the network begins to exhibit regular synchronous bursts. We thus aimed to quantify how this maturation process specifically altered neuronal depolarization waveform parameters. We performed daily time-series fluorescence imaging of neuronal cultures from DIV8 through DIV15 (Fig. 2A). Following image analysis to identify individual neurons, fluorescent traces for each ROI were normalized to the minimum fluorescence intensity (F_min_) for that cell over the duration of the acquisition period (Fig. 2B) and then converted to raster plots (Fig. 2C). At each day *in vitro* (DIV8-15) we extracted waveform parameters from the normalized fluorescence traces using time-domain analysis. Extracted parameters include average amplitude (Fig. 3A), pulse width (Fig. 3B), frequency (Fig. 3C), fall time (Fig. 3D), and rise time (Fig. 3E), as well as duty cycle (Figure S2A) and slew rates (Figure S2B). An overview of acquisition and analysis of waveform parameters is provided in Figure S3. Spike amplitude and rise time relate to the relative magnitude and rate of the rise in intracellular calcium, whereas pulse width and fall time reflect bursting duration and the rate of subsequent calcium sequestration within the cell. Frequency in this context does not reflect spike frequency, per se, as individual spikes were not distinguished during bursts; rather, frequency reflects the inverse of the inter-burst interval. For each ROI we also measured the degree of network synchronization by computing the average within-frame inter-ROI Pearson correlation coefficient^34^ (Fig. 3F). As the cultures matured, neuronal calcium transients became more synchronous, less frequent, and exhibited longer pulse widths, fall times, and rise times (representative neuronal calcium traces and raster plots are shown in Fig. 2B and C respectively; aggregate data are plotted in Fig. 3). The normalized amplitude for the cultures also trended toward an increase over time, but this effect may be due to a cumulative increase in GCaMP6f expression in each cell. Notably, even at later time points we did not see expression of GCaMp6f in non-neural subtypes, such as astrocytes (Figure S4A) or oligodendrocytes (Figure S4B), indicating that changes in amplitude or frequency were not the result of calcium signaling in other cell types. The coefficient of variation (CV) for each extracted parameter was also determined in order to gauge the inherent spatial and temporal variability in neuronal firing patterns. Variation between fields (8–22%; Fig. 3G) and within the same field imaged at different times (13–43%; Fig. 3H) was small, indicating reproducibility at the level of an individual cell through time and at the network level. However, as expected, there was a high degree of variability between individual neurons within any given acquisition frame, with inter-ROI CVs ranging from 66% to 154% (Fig. 3I), indicating a wide range of cellular response profiles.

### Validation of cortical neuron network responses to neurotransmitter receptor agonists and antagonists

**Figure 4:**
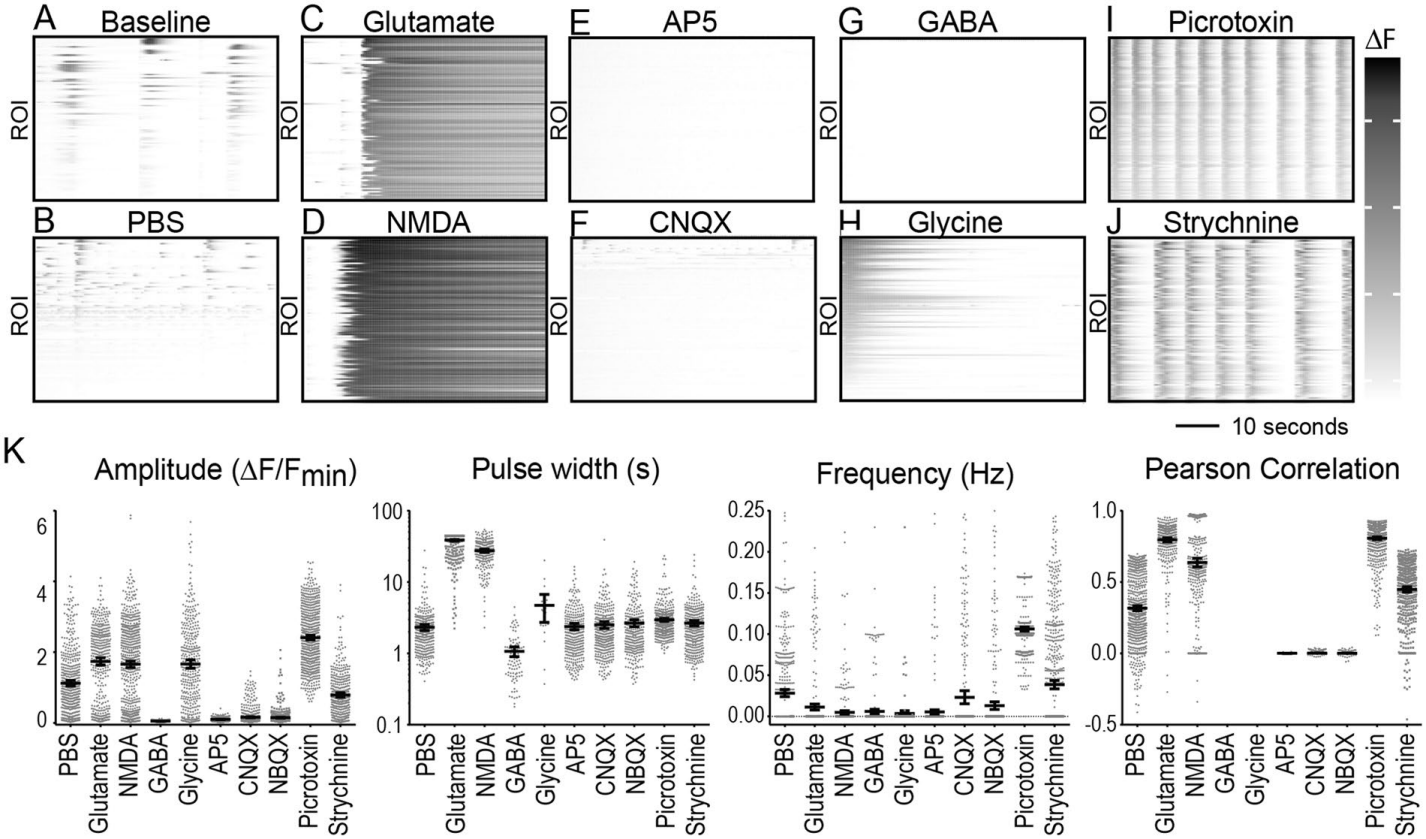
Waveform perturbation by receptor agonists and antagonists. (**A**) DIV11 cortical neurons were (**A**) left untreated or acutely treated with (**B**) PBS, (**C**) glutamate (23 μM), (**D**) NMDA (35 μM), (**E**) AP5 (29 μM), (**F**) CNQX (3 μM), (**G**) GABA (27 μM), (**H**) glycine (1.7 mM), (**I**) picrotoxin (2.4 μM), or (**J**) strychnine (6.3 μM). Representative raster plots show Δ fluorescence traces for each ROI within the optical field. Data are representative of 3–6 treatment replicates across two independent experiments. (**K**) Average amplitude, frequency, pulse width, and Pearson correlation coefficient are plotted. Error bars represent 95% CI.

To validate the automated analysis of network activity we treated semi-synchronized cultures at DIV11 with known neurotransmitter receptor agonists and antagonists to drive changes in neural activity. Excitatory activity was modulated with the glutamatergic receptor agonists glutamate and N-methyl-D-aspartate (NMDA) and the glutamatergic receptor antagonists 2,3-dihydroxy-6-nitro-7-sulfamoyl-benzo[f]quinoxaline-2,3-dione (NBQX), 6-cyano-7-nitroquinoxaline-2,3-dione (CNQX), and 2-amino-5-phosphonopentanoic acid (AP5). Inhibitory activity in the network was modulated with the glycinergic receptor agonist glycine, the glycinergic antagonist strychnine, the GABAergic receptor agonist GABA, and the GABA_A_ receptor antagonist picrotoxin. Waveform parameters including amplitude, pulse width, mean firing rate (frequency), and synchronization (Pearson correlation) (Fig. 4K), as well as duty cycle and slew rate (Figure S5) were extracted from fluorescent traces acquired following acute treatments.

Compared to untreated (Fig. 4A) or vehicle treated cells (Fig. 4B), neuronal cultures treated acutely with glutamate (23 μM; Fig. 4C) or NMDA (35 μM; Fig. 4D) exhibited sustained elevation of intracellular calcium. Waveform analysis revealed increased amplitude, increased pulse width, decreased frequency, and increased synchronization across cells, consistent with tonic activation of calcium-permeable ionotropic glutamate receptors (Fig. 4K). Blocking NMDA receptors with AP5 (29 μM) completely suppressed all semi-synchronous calcium transients observed at baseline (Fig. 4E). Waveform analysis indicated strongly decreased amplitude, frequency, and network synchrony, though the pulse width of calcium transients that did occur was unchanged (Fig. 4K). This suggests a primary role for NMDA receptors in the spontaneous calcium flux observed in our neuronal culture system. In parallel, the α-amino-3-hydroxy-5-methyl-4-isoxazolepropionic acid (AMPA) and kainate receptor antagonists CNQX (3 μM; Fig. 4F,K) and NBQX (1.3 μM; Fig. 4K) both reduced the amplitude and network synchronization of calcium signals in the neurons but did not change the pulse width and only modestly decreased the frequency of the signals (Fig. 4K). These findings indicate that both NMDA and AMPA/kainate receptors are necessary for high-amplitude, synchronized spontaneous calcium transients in the network, but NMDA receptors drive the frequency response independently of AMPA/kainate receptors.

Stimulation of inhibitory synapses in the network revealed that exogenous GABA (27 μM; Fig. 4G) essentially completely suppressed all calcium signaling (Fig. 4K) while glycine (1.7 mM; Fig. 4H) disrupted pulsatile synchrony without directly reducing signal amplitude (Fig. 4K). Blocking GABAergic receptors with picrotoxin (2.4 μM; Fig. 4I) increased signal amplitude, robustly increased the frequency of spontaneous calcium transients, and strongly synchronized the network (Fig. 4K). Likewise the glycinergic receptor antagonist strychnine (6.3 μM; Fig. 4J) synchronized the network but did not alter amplitude or frequency to the same extent as picrotoxin (Fig. 4K). These findings suggest that both GABAergic and glycinergic transmission contribute to modulating spontaneous synchronization in the network, with GABAergic receptors also contributing to suppression of signal frequency and amplitude. Overall, we conclude that our neuronal network system exhibits robust dynamic range for calcium signaling, with combinatorial input from NMDA, AMPA/kainate, glycine, and GABA receptor systems controlling the frequency and amplitude of calcium transients and establishing network synchrony parameters.

### Characterization of cortical neuronal network responses to inflammatory cytokines

**Figure 5:**
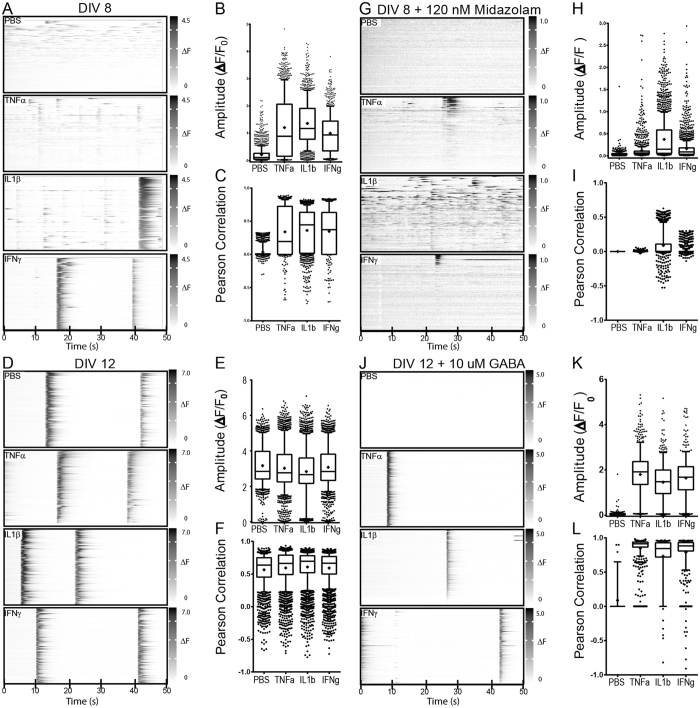
Cytokine treatment alters neuronal firing kinetics and increases network synchronization. Representative raster plots are shown for DIV 8 (**A**,**G**) or DIV 12 (**D**,**J**) cortical neurons treated for 24 hours with 100 ng /mL TNF-α, 100 ng/ mL IFN-γ, 10 ng/mL IL-1β or vehicle alone (PBS). After 24 hours of cytokine stimulation, cells were treated acutely with 120 nM midazolam (**G**–**I**) or 10 μM GABA (**J**–**L**) prior to signal acquisition. Average amplitude and Pearson correlation coefficient values are plotted for DIV 8 neurons prior to (**B**,**C**) and after treatment with 120 nM midazolam (**H**,**I**) as well as for DIV 12 neurons prior to (**E**,**F**) and after treatment with 10 uM GABA (**K**,**L**) for all ROIs across 6 wells for each of the indicated conditions. Box plots show median (line), average (+), and interquartile range; error bars represent 10–90% range with outliers shown. Data are representative of 2–3 independent experiments.

The dynamic range and balance of excitatory and inhibitory synaptic function demonstrated in our system suggested that it could be used to determine the impact of inflammatory cytokines on neural network properties. Cultures at DIV8 (Fig. 5A,G) and DIV12 (Fig. 5D,J) were treated for 24 hr with TNFα (100 ng/mL), IL1β (10 ng/mL), or IFNγ (100 ng/mL) and spontaneous calcium transients were compared to vehicle-treated (PBS) cells. As shown above, vehicle-treated cultures at DIV8 exhibited unsynchronized low amplitude calcium signals. All three cytokines induced both a large increase in signal amplitude (Fig. 5A,B) (F(3,4873) = 632.53, p < 0.0001; all 3 cytokines different from PBS at p < 0.0001 by Steel method vs control; IL1b vs PBS, d = 1.26; TNFa vs PBS, d = 1.09; IFNg vs PBS, d = 0.85) and an increase in neuronal synchronization (Fig. 5A,C) (F(3,5345) = 36.63, p < 0.0001; all 3 cytokines different from PBS at p < 0.0001 by Steel method vs control; IL1b vs PBS, d = 1.02; TNFa vs PBS, d = 0.95; IFNg vs PBS, d = 0.98). In contrast, cytokine treatment of DIV12 cultures, which already exhibited high amplitude, highly synchronized calcium signals, resulted in only small, biologically insignificant changes in amplitude (Fig. 5D,E,F) (F(3,10824) = 39.55, p < 0.0001; IL1β and TNFα different from PBS at p < 0.0001, IFNγ at P = 0.0215 by Steel method vs control; IL1b vs PBS, d = 0.29; TNFa vs PBS, d = 0.17; IFNg vs PBS, d = 0.21) and synchronization (F(3,10691) = 11.15, p < 0.0001; all 3 cytokines different from PBS at p < 0.0001 by Steel method vs control; IL1b vs PBS, d = 0.17; TNFa vs PBS, d = 0.11; IFNg vs PBS, d = 0.11). Likewise, no significant changes in baseline activity of mature DIV 12 cultures were observed using cytokine concentrations that were up to 1000x lower than in initial experiments (Figure S6).

In order to probe the role of inhibitory synaptic function in the effect observed at DIV8 we treated cultures for 24 hr with cytokines, as above, and then acutely treated with midazolam (120 nM), a benzodiazepine that enhances endogenous GABA activity, immediately before imaging. While midazolam robustly suppressed signal amplitude and synchronization in the vehicle-treated cells, it did not have the same effect in the cytokine-treated cultures (Fig. 5G,H,I). While signal amplitudes (Fig. 5H vs 5B) and synchronization (Fig. 5I vs 5C) were reduced, there was persistence of significant network activity and synchrony in the IL1β-treated cells (amplitude: F(3,5956) = 310.51, p < 0.0001; all 3 cytokines different from PBS at p < 0.0001 by Steel method vs control; IL1b vs PBS, d = 0.99; TNFa vs PBS, d = 0.23; IFNg vs PBS, d = 0.36; synchrony: F(3,4432) = 147.95, p < 0.0001; IL1β different from PBS at p = 0.0021, TNFα not different at p = 0.2717, IFNγ not different at p = 0.1939 by Steel method vs control; IL1b vs PBS, d = 0.68; TNFa vs PBS, d = 0.01; IFNg vs PBS, d = 0.13). All three cytokine-treated conditions exhibited populations of almost completely silenced cells and a population that was blunted but still active (Fig. 5H), though only the IL1β-treated cells exhibited a biologically significant effect size measurement. This finding suggests that cytokine treatment, and especially IL1β, drives increased amplitude and network synchronization in DIV8 cells, at least in part, by reducing GABAergic input at some synapses.

Finally, despite no apparent effect of cytokines on network properties at DIV12, we treated these cells acutely with 10 μM GABA and assessed changes in calcium signaling. As expected, based on Fig. 4, GABA treatment completely silenced the vehicle-treated cultures (Fig. 5J,K). Surprisingly, however, this concentration of GABA only slightly reduced the signal amplitude in the cytokine-treated cells (Fig. 5J,K) (F(3,2752) = 546.03, p < 0.0001; all 3 cytokines different from PBS at p < 0.0001 by Steel method vs control; IL1b vs PBS, d = 1.37; TNFa vs PBS, d = 1.71; IFNg vs PBS, d = 1.56) and the level of network synchronization actually increased in these cells in the presence of GABA (Fig. 5L) (F(3,2118) = 104.45, p < 0.0001; all 3 cytokines different from PBS at p < 0.0001 by Steel method vs control; IL1b vs PBS, d = 2.34; TNFa vs PBS, d = 2.77; IFNg vs PBS, d = 2.56). This finding suggests that cytokine treatment of mature neurons, while having no obvious effect on baseline activity, strongly impacts GABAergic tone in the network. Changes in expression or localization of excitatory neurotransmitters and their receptors have been previously implicated in the hyperexcitability of neural networks following exposure to inflammatory factors^35^. However, we did not observe significant changes in network activity in cytokine treated neurons following acute stimulation with 5 μM glutamate (data not shown). Rather, our findings suggest that cytokine mediated disruption of inhibitory GABAergic signaling may likewise contribute to cortical hyperexcitability in both developing and mature neural networks.

## Discussion

Here we describe methods for quickly analyzing firing rate, synchrony and waveform parameters for spontaneous and induced calcium transients in primary neurons. Importantly, our approach relies on widely available software and hardware tools, eliminating many of the significant technical obstacles of the more sophisticated imaging platforms^36^. We use a commercially available genetically encoded calcium indicator that explicitly excludes confounding signals derived from non-neuronal cells in mixed neural cell *in vitro* preparations. This genetically encoded indicator also reduces signal loss due to photobleaching during longitudinal sampling when compared to imaging with calcium indicator dyes allowing for serial imaging of individual cultures. Together, these advantages provide substantial improvement upon previous reports that relied on calcium indicator dyes^37^. These genetically encoded indicators are state-of-the-art for *in vivo* imaging of neuronal activity^38–40^ and provide exceptional power for *in vitro* analyses. A principle advantage of high-content wide-field calcium imaging is that it allows simultaneous recording from hundreds of neurons while retaining the ability to distinguish calcium fluxes at the single cell level, respectively overcoming limitations of traditional patch clamp and more recent microelectrode array approaches^19^. However, compared to these other approaches, the sampling rate of calcium imaging is more limited. For example, patch clamp methods and microelectrode array methods for measuring membrane and extracellular field potentials often achieve sampling rates in excess of 10 KHz; calcium imaging methods have more meager frame rates typically ranging from 1–100 Hz. Additionally, calcium imaging, in general, and especially automated approaches as described here, rely principally on the detection of broad somatic calcium transients rather than rapid dendritic calcium transients. Thus, calcium imaging-based approaches have less utility in detecting fast-spiking neuronal action potentials, instead resolving neuronal bursting events as prolonged calcium spikes. It should also be noted that subthreshold depolarization events can lead to transient increases in intracellular calcium. This limits the utility of frequency domain analysis for which Fourier transformations do not yield easily interpretable results. Thus, time-domain analysis of wave form parameters becomes more useful for assessing changes in the analysis of bursting and tonic activity in neurons assessed by calcium imaging.

Our studies focus on spike amplitude, positive pulse width, and burst frequency. As mentioned before, these waveform parameters indicate the magnitude of the rise in intracellular calcium, average burst duration, and the inter-burst interval, respectively. In addition, we provide methods for extracting average positive pulse rise time, fall time, and slew rate from normalized fluorescence traces of individual regions of interest. These parameters define the shape of calcium transient waves, and therefore, when used in conjunction with the other measures, will be useful for segregating distinct neuronal populations based on underlying factors such as surface to volume ratio and calcium buffering capacity, which contribute to the waveform of calcium transients^41^.

Another benefit afforded by the use of genetically encoded calcium indicators is longitudinal tracking of neuronal network activity in cultured primary neurons as they mature or longitudinal tracking of pre- and post-treatment effects as well as wash-out effects. We have shown using our current approaches that cultured primary cortical neurons develop more synchronous spontaneous activity as they mature with increasing average pulse widths that are indicative of longer spike bursts. These techniques could be used to study how specific genetic deficiencies alter neuronal development and network maturation *in vitro*, especially regarding genes for which genetically penetrant knock out animals do not survive to adulthood. Furthermore, the analytical approaches described herein could be readily adapted to or combined with other recently described methods for analysis of organotypic slice cultures which would provide tissue context for studies assessing how specific factors affect developing neural networks^42^. In particular, it has been proposed that neuroinflammatory events at critical periods during development could disturb normal network maturation, leading to lasting effects on spontaneous network activity^43–48^.

It is interesting that we observed differential responsiveness of neural networks to inflammatory cytokines based on maturation state. This may be partly explained by the observed difference in the baseline state of the neural networks (i.e. more mature cultures showed higher amplitude and more synchronous calcium transient under baseline conditions). However, the literature also suggests that the responsiveness of neuronal and glial cells to inflammatory or noxious stimuli changes during development, with more immature cells often showing exaggerated or inverse responses^49–52^. For example, in hippocampal neuron cultures IFNγ was shown to increase the frequency of AMPA receptor dependent excitatory post synaptic currents (EPSCs) and further increases in EPSCs were observed when treatment was initiated at an immature stage^51^. In astrocytes it was shown that when cells are stressed by treatment with unconjugated bilirubin they secrete TNFα and glutamate and that secretion of these factors is further elevated when cells are treated at earlier days *in vitro* ^50^. Similarly, in models of hypoxia-ischemia microglial activation, proliferation, and production of proinflammatory cytokines is heightened in neonates compared to juveniles^52^.

Mechanistically, several factors are thought to contribute to these differences. First, during development widespread synapse maturation involves intricate and multicellular signaling events that lead to synapse stabilization, pruning, and synaptic scaling. Disruption of these processes at any level may derail subsequent developmental processes leading to network dysfunction. Second, the expression profile of immune receptors and pattern recognition receptors is different in the developing and adult brain^53^ (reviewed in ref. 48), and therefore these cells may be poised for exaggerated responses to inflammatory stimuli. Lastly, the expression of anti-inflammatory and neuroprotective factors is known to contribute to the immune privilege of the adult brain, but the extent to which these factors are expressed in developing brain cells, especially *in vitro*, is unknown. In line with this, a recent study in rats found that TNFα-inducible protein 6 (TNFaip6) is increasingly expressed from P1-P15, and when administered to neonates it mitigated inflammation-induced brain injury in an LPS injection model^54^. Thus, reduced levels of anti-inflammatory or neuroprotective proteins in the developing brain, and concomitantly in the less mature neural cell cultures, may explain the increased sensitivity of these cells to pro-inflammatory cytokines.

We also found that treatment with pro-inflammatory cytokines resulted in reduced sensitivity of neurons to exogenous GABA receptor agonists regardless of culture maturation state. Loss of GABAergic tone is known to contribute to network hyperexcitability and neuroinflammation at any stage of life has been implicated in neuronal network disruption and is thought to drive cognitive impairment and abnormal EEG findings (including seizures) in a variety of primary neuroinflammatory and CNS autoimmune diseases. Network hyperexcitability has also been described in the aging brain, which could be compounded by acute neuroinflammatory insult. Moreover, the cumulative effect of mild neuroinflammatory episodes on network hyperexcitability in the aging brain remains unknown. Chief among the factors that have been implicated in eliciting neuronal hyperexcitability are IL1β and TNFα. These cytokines have been shown to be elevated in epileptic tissues and rodent models of epilepsy^55–57^, as well as in serum and plasma in patients with epilepsy^58–62^, and they have allelic associations with risk for epilepsy^63, 64^ and febrile seizures^65, 66^. TNFα and IL1β have also been implicated in synaptic scaling, neuronal excitability, neurite outgrowth, neuronal maturation, and seizure susceptibility in cultured cells^49, 67–70^, organotypic slice cultures^71^ and rodent kindling models of epilepsy^72–75^. Furthermore, anti-epileptic drugs^76–78^ and temporal lobectomy^79^ have been shown to reduce serum levels of these inflammatory cytokines, which may partially explain treatment efficacy.

We found that these cytokines, along with IFNγ, another proinflammatory cytokine, increased the amplitude and synchrony of neuronal calcium transients. These findings concur with a recent report showing that LPS stimulation increased microglial TNFα secretion and thereby caused an increase in spike burst duration in neuronal cultures within 5 hours, as assessed by multielectrode array^68^. However, TNFα alone induced neuronal excitability changes in this system that were distinct from those induced by LPS-activated microglia, suggesting that other factors secreted by LPS-activated microglia, such as IL1β, could be contributing to the emergence of long-burst events^68^. In line with this, it was recently shown that IL1β reduces GABA-A mediated currents in resected hippocampal tissue from patients with temporal lobe epilepsy^80^. Therefore, though it is possible that a single inflammatory cytokine and signal transduction pathway is responsible for the observed effects on network function, it is more likely that each of these cytokines triggers the initiation of a common web of interconnected signaling cascades in neurons, astrocytes, and microglia leading to circuit disruption. Indeed, upon injury or activation both astrocytes and microglia are capable of secreting TNFα, IL1β and IFNγ. Moreover, these factors are capable of reciprocal induction and synergistic effects^81–85^. For example, IFNγ and TNFα have been shown to promote an M1-like fate in microglia leading to microglial secretion of TNFα and IL1β^86–88^. Likewise, TNFα has been shown to promote astrocyte secretion of IFNγ^89^. In addition to their role in inflammatory processes, these cytokines have also been shown to play key roles in normal brain development. Therefore, abnormally elevated levels of these cytokines during critical periods likely derail neurodevelopment leading to brain dysfunction or latent susceptibility to inflammation triggered brain injury later in life (reviewed in ref. 90). In summary, inflammation is thought to play a key role in many developmental brain disorders, and thus a better of understanding of developmental differences in the responsiveness of neurons and glia to inflammatory insults will aid in the prevention and treatment of these disorders.

## Methods

### Cortical neuron cultures

Tissue-culture treated vessels were coated with 0.5 mg/mL poly-ornithine in 100 mM borate buffer. Cortical neurons were prepared from C57BL6 mouse embryonic day 15 (E15) pups as previously described^91^. Briefly, the upper halves of the cerebral cortices were dissected and meninges were removed and washed in Hank’s Balanced Salt Solution (HBSS). The tissue was washed in neuron plating media, which contained high-glucose Dulbecco’s Modified Eagle Medium (DMEM) with glutamine, supplemented with 10% bovine calf serum and 10% F12. Cortices were digested in 2 mg/mL papain in HBSS at 37 °C for 15 minutes, triturated with 5 ml serological pipet, p1000 pipet tip, glass Pasteur pipet and finally fire polished glass Pasteur pipet until cortices were roughly dissociated into a single cell suspension. Cells were centrifuged at 400 g for 4 min and seeded at 4.5 × 10^5^ cells per cm^2^ on poly-ornithine coated plates. Three hours after seeding, the cortical neurons were fed with 2 volumes neuron feed media containing neurobasal media supplemented with 2% B27, 1% Glutamax, and 100 U/mL penicillin and streptomycin. During the first 48 hours cells were given 1 ng/ml brain derived neurotrophic factor (BDNF) and 10 ng/ml insulin-like growth factor 1 (IGF1). Subsequently, neurons were maintained in neuron feed media by changing half media volume every 2 days.

### Cell treatments

Recombinant-murine interferon gamma (RnD Systems), tumor necrosis factor-alpha (Prospec), and interleukin 1 beta (Prospec) were aliquoted at 1000x stocks and stored at −20C. All neuroactive compounds including gamma-aminobutyric acid (GABA), N-methyl-D-aspartate (NMDA), 1,2,3,4-Tetrahydro-6-nitro-2,3-dioxo-benzo[f]quinoxaline-7-sulfonamide (NBQX), 6-Cyano-7-nitroquinoxaline-2,3-dione (CNQX), glutamate, glycine, strychnine, picrotoxin, and D(−)−2-Amino-5-phosphonopentanoic acid (AP5) were purchased from Sigma. All factors were diluted to 5x working solutions in pre-warmed neuron feed media and further diluted 5 fold upon addition to neuronal cultures.

### Morphological and immunocytochemical analysis

Neuronal cultures were lightly fixed in 2% paraformaldehyde for 25 minutes at room temperature, washed, and blocked with 1% bovine serum albumin, 0.1% Triton X-100, 8% donkey serum in PBS. Antibodies against GFAP (GA5, EMD Millipore), NeuN (A60, EMD Millipore), MAP2 (AP20, EMD Millipore), GAD67 (IG10.2, EMD Millipore) Olig2 (211F1.1, EMD Millipore), TBR1 (polyclonal chicken AB2261, EMD Millipore), and Iba1 (polyclonal rabbit, Wako Chemicals) were diluted 1:200–1000 in blocking buffer and samples were stained overnight at 4 C. Cells were repeatedly washed with PBS and stained for 2 hours at room temperature with AlexaFluor® 488 and AlexaFluor® 568 conjugated donkey secondary antibodies diluted 1:200–500 in blocking buffer. Cells were counterstained with DAPI diluted in PBS 1:1000 and imaged with an LSM 780 confocal microscope. Fluorescent micrographs were exported as.tif files from Zen software (Zeiss).

### Transcriptional analysis

Cortical neuron lysates were dissociated and homogenized using QIAShredder (Qiagen), following the manufacturer’s instructions. RNA was isolated using RNeasy micro plus kit (Qiagen) and genomic DNA was excluded using gDNA eliminator spin column. Relative expression of the indicated neurotransmitter receptor subunits was quantitated relative to the house keeping gene UROD. Gene specific primers and probes (Supplementary Table I) were selected using universal probe library assay design center (Roche) and 20 μL reactions using Universal probe master mix (Roche) were carried out following the manufacturer’s instructions. Optimal probe concentrations were determined empirically.

### Calcium imaging

For calcium imaging experiments, neurons were infected at plating with the adeno-associated viral vector AAV1.Syn.GCaMP6f.WPRE.SV40 (University of Pennsylvania Vector Core) at a multiplicity of infection (MOI) of 2000. Fluorescence imaging was performed on an LSM 5 Live Microscope equipped with a 10x long working distance apochromatic objective, 100 mW 488 nm laser diode, AxioCam digital microscope camera, and stage-mounted environmental chamber to maintain cells at 37 C, 5% CO2 and 100% humidity. Cells were allowed to equilibrate prior to imaging. Time series images were acquired at a sampling rate of 5–10 Hz with a spatial resolution of 1024 × 1024 pixels over 1–10 minutes epochs using Zen software (Zeiss) to reduce photo-bleaching.

### Image segregation and analysis

Maximum intensity projections (MIPs) of time series acquisitions were exported from Zen and loaded into ImageJ for automated image segregation using a scripted macro. Briefly, images were converted to 8-bit, contrast-enhanced, and converted to binary by thresholding. Single neuron regions of interests (ROIs) were extracted from background subtracted binary images by sequentially performing the following functions: enhance contrast, remove-outliers, watershed, erode, dilate and analyze particles. Subsequently, ΔF traces were measured for each neuron using the multi-measure function, and normalized by subtracting F_min_ and dividing by F_min_. Matrices of ΔF traces for all firing neurons within an optical field were analyzed in Matlab using signal processing toolbox to extract: frequency, amplitude, slew rate, rise time, and fall time. For Pearson correlation, traces were thresholded to ΔF > 0.15 to exclude baseline fluctuations from correlation indices. A Pearson Correlation matrix was calculated for all neuron pairs and data are presented as average Pearson correlation of a given neuron across all pairings. Representative raster plots were generated from fluorescence traces using Gitools^92^. Further details of software scripts are provided in supplementary materials.

### Animal care and use

All animal experiments were approved by the Mayo Clinic Institutional Animal Care and Use Committee in accordance with National Institutes of Health guidelines.

### Statistical analysis

Analyses were performed in GraphPad Prism 6.02 (GraphPad Software, Inc.), the SigmaStat component of SigmaPlot (Systat Software, Inc.), or JMP Pro 12 (SAS Institute Inc.). α = 0.05 and β = 0.2 were established a priori. Post hoc power analysis was performed for all experiments and significance was only considered when power ≥ 0.8. Normality was assessed with the Lilliefors modification of the Kolmogorov-Smirnov goodness-of-fit test and non-parametric tests were applied as warranted. Least square linear and non-linear regression analyses were performed using GraphPad Prism 6.02, using the robust regression and outlier removal method (Q = 1%). For data in Fig. 5 the least squares ANOVA was calculated and the Steel method vs control was used for pairwise comparison of treatments to vehicle. The effect sizes for differences in Fig. 5 were estimated using Cohen’s d on the least squares fit^93^. Multiplicity-corrected p values are reported as warranted. Graphical results are presented as means; error bars represent the 95% confidence interval, except Fig. 5, in which error bars represent 10–90% range. Descriptive statistics are provided as appropriate and statistical values are reported following Curran-Everett guidelines^94^.

## Electronic supplementary material

Supplementary Materials

## References

[1] Centonze D (2010). The link between inflammation, synaptic transmission and neurodegeneration in multiple sclerosis. Cell Death Differ.

[2] Musella A, Mandolesi G, Mori F, Gentile A, Centonze D (2016). Linking synaptopathy and gray matter damage in multiple sclerosis. Mult Scler.

[3] Mandolesi G (2015). Synaptopathy connects inflammation and neurodegeneration in multiple sclerosis. Nat Rev Neurol.

[4] Vincent A, Lang B, Kleopa KA (2006). Autoimmune channelopathies and related neurological disorders. Neuron.

[5] Kenney-Jung DL (2016). Febrile infection-related epilepsy syndrome treated with anakinra. Ann Neurol.

[6] Saitoh M (2016). Cytokine-related and sodium channel polymorphism as candidate predisposing factors for childhood encephalopathy FIRES/AERRPS. J Neurol Sci.

[7] Diamond ML (2014). IL-1beta associations with posttraumatic epilepsy development: a genetics and biomarker cohort study. Epilepsia.

[8] Karve IP, Taylor JM, Crack PJ (2016). The contribution of astrocytes and microglia to traumatic brain injury. Br J Pharmacol.

[9] Vezzani A, Viviani B (2015). Neuromodulatory properties of inflammatory cytokines and their impact on neuronal excitability. Neuropharmacology.

[10] Vezzani A, Friedman A, Dingledine RJ (2013). The role of inflammation in epileptogenesis. Neuropharmacology.

[11] Murashima YL, Suzuki J, Yoshii M (2008). Role of cytokines during epileptogenesis and in the transition from the interictal to the ictal state in the epileptic mutant EL mouse. Gene Regul Syst Bio.

[12] Alyu F, Dikmen M (2017). Inflammatory aspects of epileptogenesis: contribution of molecular inflammatory mechanisms. Acta Neuropsychiatr.

[13] Eldawlatly S, Oweiss KG (2014). Temporal precision in population-but not individual neuron-dynamics reveals rapid experience-dependent plasticity in the rat barrel cortex. Front Comput Neurosci.

[14] Kuang SY (2015). Prolonging life in chick forebrain-neuron culture and acquiring spontaneous spiking activity on a microelectrode array. Biotechnol Lett.

[15] Johnson LJ (2012). A novel high electrode count spike recording array using an 81,920 pixel transimpedance amplifier-based imaging chip. J Neurosci Methods.

[16] Boehler MD, Wheeler BC, Brewer GJ (2007). Added astroglia promote greater synapse density and higher activity in neuronal networks. Neuron Glia Biol.

[17] Kaneko H, Tamura H, Suzuki SS (2007). Tracking spike-amplitude changes to improve the quality of multineuronal data analysis. IEEE Trans Biomed Eng.

[18] Kuang SY (2016). How Microelectrode Array-Based Chick Forebrain Neuron Biosensors Respond to Glutamate NMDA Receptor Antagonist AP5 and GABAA Receptor Antagonist Musimol. Sens Biosensing Res.

[19] Cornelissen F (2013). Quantitation of chronic and acute treatment effects on neuronal network activity using image and signal analysis: toward a high-content assay. J Biomol Screen.

[20] Amin H (2016). Electrical Responses and Spontaneous Activity of Human iPS-Derived Neuronal Networks Characterized for 3-month Culture with 4096-Electrode Arrays. Front Neurosci.

[21] Lonardoni D (2015). High-density MEA recordings unveil the dynamics of bursting events in Cell Cultures. Conf Proc IEEE Eng Med Biol Soc.

[22] Matsuda E (2013). Analysis of neuronal cells of dissociated primary culture on high-density CMOS electrode array. Conf Proc IEEE Eng Med Biol Soc.

[23] Yada Y, Kanzaki R, Takahashi H (2016). State-Dependent Propagation of Neuronal Sub-Population in Spontaneous Synchronized Bursts. Front Syst Neurosci.

[24] Yada Y (2016). Development of neural population activity toward self-organized criticality. Neuroscience.

[25] Ferrea E (2012). Large-scale, high-resolution electrophysiological imaging of field potentials in brain slices with microelectronic multielectrode arrays. Front Neural Circuits.

[26] Robel S, Sontheimer H (2016). Glia as drivers of abnormal neuronal activity. Nat Neurosci.

[27] Chen TW (2013). Ultrasensitive fluorescent proteins for imaging neuronal activity. Nature.

[28] Hevner RF (2001). Tbr1 regulates differentiation of the preplate and layer 6. Neuron.

[29] Sahara S, Yanagawa Y, O’Leary DD, Stevens CF (2012). The fraction of cortical GABAergic neurons is constant from near the start of cortical neurogenesis to adulthood. J Neurosci.

[30] Fletcher TL, Cameron P, De Camilli P, Banker G (1991). The distribution of synapsin I and synaptophysin in hippocampal neurons developing in culture. J Neurosci.

[31] Salemi G, Ferraro D, Savettieri G (1990). Triiodothyronine accelerates the synthesis of synapsin I in developing neurons from fetal rat brain cultured in a synthetic medium. Neurochem Res.

[32] Di Liegro I (1995). Expression of synapsin I gene in primary cultures of differentiating rat cortical neurons. Neurochem Res.

[33] Patel TP, Man K, Firestein BL, Meaney DF (2015). Automated quantification of neuronal networks and single-cell calcium dynamics using calcium imaging. J Neurosci Methods.

[34] Ferguson MA, Anderson JS (2012). Dynamical stability of intrinsic connectivity networks. Neuroimage.

[35] Golde S, Chandran S, Brown GC, Compston A (2002). Different pathways for iNOS-mediated toxicity *in vitro* dependent on neuronal maturation and NMDA receptor expression. J Neurochem.

[36] Yang W, Yuste R (2017). *In vivo* imaging of neural activity. Nat Methods.

[37] Tibau E, Valencia M, Soriano J (2013). Identification of neuronal network properties from the spectral analysis of calcium imaging signals in neuronal cultures. Front Neural Circuits.

[38] Dana H (2014). Thy1-GCaMP6 transgenic mice for neuronal population imaging *in vivo*. PLoS One.

[39] Dana, H. *et al*. Sensitive red protein calcium indicators for imaging neural activity. *Elife* **5**, doi:10.7554/eLife.12727 (2016). 10.7554/eLife.12727 PMC4846379 27011354

[40] Moretti C, Antonini A, Bovetti S, Liberale C, Fellin T (2016). Scanless functional imaging of hippocampal networks using patterned two-photon illumination through GRIN lenses. Biomed Opt Express.

[41] Ruffinatti FA, Gilardino A, Lovisolo D, Ferraro M (2013). Spatial wavelet analysis of calcium oscillations in developing neurons. PLoS One.

[42] Perez-Ortega J (2016). Pathophysiological signatures of functional connectomics in parkinsonian and dyskinetic striatal microcircuits. Neurobiol Dis.

[43] Kramer K (2012). TNF-overexpression in Borna disease virus-infected mouse brains triggers inflammatory reaction and epileptic seizures. PLoS One.

[44] Lee RH (2010). Neurodevelopmental effects of chronic exposure to elevated levels of pro-inflammatory cytokines in a developing visual system. Neural Dev.

[45] Anderson V, Catroppa C, Morse S, Haritou F, Rosenfeld J (2005). Functional plasticity or vulnerability after early brain injury?. Pediatrics.

[46] Hessen E, Nestvold K, Anderson V (2007). Neuropsychological function 23 years after mild traumatic brain injury: a comparison of outcome after paediatric and adult head injuries. Brain Inj.

[47] Hagberg H, Gressens P, Mallard C (2012). Inflammation during fetal and neonatal life: implications for neurologic and neuropsychiatric disease in children and adults. Ann Neurol.

[48] Hagberg H (2015). The role of inflammation in perinatal brain injury. Nat Rev Neurol.

[49] Bernardino L (2008). Tumor necrosis factor-alpha modulates survival, proliferation, and neuronal differentiation in neonatal subventricular zone cell cultures. Stem Cells.

[50] Falcao AS, Fernandes A, Brito MA, Silva RF, Brites D (2006). Bilirubin-induced immunostimulant effects and toxicity vary with neural cell type and maturation state. Acta Neuropathol.

[51] Vikman KS, Owe-Larsson B, Brask J, Kristensson KS, Hill RH (2001). Interferon-gamma-induced changes in synaptic activity and AMPA receptor clustering in hippocampal cultures. Brain Res.

[52] Ferrazzano P (2013). Age-dependent microglial activation in immature brains after hypoxia- ischemia. CNS Neurol Disord Drug Targets.

[53] Shi H, Gabarin N, Hickey E, Askalan R (2013). TLR-3 receptor activation protects the very immature brain from ischemic injury. J Neuroinflammation.

[54] Bertling F (2016). Tumor necrosis factor-inducible gene 6 protein: A novel neuroprotective factor against inflammation-induced developmental brain injury. Exp Neurol.

[55] Ravizza T (2006). The IL-1beta system in epilepsy-associated malformations of cortical development. Neurobiol Dis.

[56] Ravizza T, Vezzani A (2006). Status epilepticus induces time-dependent neuronal and astrocytic expression of interleukin-1 receptor type I in the rat limbic system. Neuroscience.

[57] Vezzani A (2002). Functional role of inflammatory cytokines and antiinflammatory molecules in seizures and epileptogenesis. Epilepsia.

[58] Mao LY (2013). Interictal interleukin-17A levels are elevated and correlate with seizure severity of epilepsy patients. Epilepsia.

[59] Gao, F. *et al*. Alteration of plasma cytokines in patients with active epilepsy. *Acta Neurol Scand*, doi:10.1111/ane.12665 (2016). 10.1111/ane.12665 27593211

[60] Rosa DV (2016). Circulating CD4 and CD8 T cells expressing pro-inflammatory cytokines in a cohort of mesial temporal lobe epilepsy patients with hippocampal sclerosis. Epilepsy Res.

[61] Sinha S, Patil SA, Jayalekshmy V, Satishchandra P (2008). Do cytokines have any role in epilepsy?. Epilepsy Res.

[62] Hulkkonen J (2004). The balance of inhibitory and excitatory cytokines is differently regulated *in vivo* and *in vitro* among therapy resistant epilepsy patients. Epilepsy Res.

[63] Saghazadeh A, Gharedaghi M, Meysamie A, Bauer S, Rezaei N (2014). Proinflammatory and anti-inflammatory cytokines in febrile seizures and epilepsy: systematic review and meta-analysis. Rev Neurosci.

[64] Jin L (2003). Association analysis of a polymorphism of interleukin 1 beta (IL-1 beta) gene with temporal lobe epilepsy in a Chinese population. Epilepsia.

[65] Dube C, Brunson KL, Eghbal-Ahmadi M, Gonzalez-Vega R, Baram TZ (2005). Endogenous neuropeptide Y prevents recurrence of experimental febrile seizures by increasing seizure threshold. J Mol Neurosci.

[66] Haspolat S (2005). Interleukin-1alpha, interleukin-1beta, and interleukin-1Ra polymorphisms in febrile seizures. J Child Neurol.

[67] Barish ME, Mansdorf NB, Raissdana SS (1991). Gamma-interferon promotes differentiation of cultured cortical and hippocampal neurons. Dev Biol.

[68] Gullo F (2014). Atypical “seizure-like” activity in cortical reverberating networks *in vitro* can be caused by LPS-induced inflammation: a multi-electrode array study from a hundred neurons. Front Cell Neurosci.

[69] Han T (2016). Seizure induced synaptic plasticity alteration in hippocampus is mediated by IL-1beta receptor through PI3K/Akt pathway. Am J Transl Res.

[70] Klapal L, Igelhorst BA, Dietzel-Meyer ID (2016). Changes in Neuronal Excitability by Activated Microglia: Differential Na(+) Current Upregulation in Pyramid-Shaped and Bipolar Neurons by TNF-alpha and IL-18. Front Neurol.

[71] Bernardino L, Ferreira R, Cristovao AJ, Sales F, Malva JO (2005). Inflammation and neurogenesis in temporal lobe epilepsy. Curr Drug Targets CNS Neurol Disord.

[72] Balosso S (2008). A novel non-transcriptional pathway mediates the proconvulsive effects of interleukin-1beta. Brain.

[73] Maroso M (2011). Interleukin-1beta biosynthesis inhibition reduces acute seizures and drug resistant chronic epileptic activity in mice. Neurotherapeutics.

[74] Maroso M (2011). Interleukin-1 type 1 receptor/Toll-like receptor signalling in epilepsy: the importance of IL-1beta and high-mobility group box 1. J Intern Med.

[75] Weinberg MS, Blake BL, McCown TJ (2013). Opposing actions of hippocampus TNFalpha receptors on limbic seizure susceptibility. Exp Neurol.

[76] Gomez CD, Buijs RM, Sitges M (2014). The anti-seizure drugs vinpocetine and carbamazepine, but not valproic acid, reduce inflammatory IL-1beta and TNF-alpha expression in rat hippocampus. J Neurochem.

[77] Christensen KV (2010). Levetiracetam attenuates hippocampal expression of synaptic plasticity-related immediate early and late response genes in amygdala-kindled rats. BMC Neurosci.

[78] Sitges M, Gomez CD, Aldana BI (2014). Sertraline reduces IL-1beta and TNF-alpha mRNA expression and overcomes their rise induced by seizures in the rat hippocampus. PLoS One.

[79] Quirico-Santos T (2013). Resection of the epileptogenic lesion abolishes seizures and reduces inflammatory cytokines of patients with temporal lobe epilepsy. J Neuroimmunol.

[80] Roseti C (2015). GABAA currents are decreased by IL-1beta in epileptogenic tissue of patients with temporal lobe epilepsy: implications for ictogenesis. Neurobiol Dis.

[81] Barcia C (2011). IFN-gamma signaling, with the synergistic contribution of TNF-alpha, mediates cell specific microglial and astroglial activation in experimental models of Parkinson’s disease. Cell Death Dis.

[82] Wang WY, Tan MS, Yu JT, Tan L (2015). Role of pro-inflammatory cytokines released from microglia in Alzheimer’s disease. Ann Transl Med.

[83] Liddelow SA (2017). Neurotoxic reactive astrocytes are induced by activated microglia. Nature.

[84] Chung IY, Benveniste EN (1990). Tumor necrosis factor-alpha production by astrocytes. Induction by lipopolysaccharide, IFN-gamma, and IL-1 beta. J Immunol.

[85] Zhao J, O’Connor T, Vassar R (2011). The contribution of activated astrocytes to Abeta production: implications for Alzheimer’s disease pathogenesis. J Neuroinflammation.

[86] Hanisch UK (2002). Microglia as a source and target of cytokines. Glia.

[87] Nguyen VT, Benveniste EN (2002). Critical role of tumor necrosis factor-alpha and NF-kappa B in interferon-gamma -induced CD40 expression in microglia/macrophages. J Biol Chem.

[88] Mangano EN (2012). Interferon-gamma plays a role in paraquat-induced neurodegeneration involving oxidative and proinflammatory pathways. Neurobiol Aging.

[89] Xiao BG, Link H (1998). IFN-gamma production of adult rat astrocytes triggered by TNF-alpha. Neuroreport.

[90] Meyer U (2013). Developmental neuroinflammation and schizophrenia. Prog Neuropsychopharmacol Biol Psychiatry.

[91] Sauer BM, Schmalstieg WF, Howe CL (2013). Axons are injured by antigen-specific CD8(+) T cells through a MHC class I- and granzyme B-dependent mechanism. Neurobiol Dis.

[92] Perez-Llamas C, Lopez-Bigas N (2011). Gitools: analysis and visualisation of genomic data using interactive heat-maps. PLoS One.

[93] Nakagawa S, Cuthill IC (2007). Effect size, confidence interval and statistical significance: a practical guide for biologists. Biol Rev Camb Philos Soc.

[94] Curran-Everett D, Benos DJ (2007). Guidelines for reporting statistics in journals published by the American Physiological Society: the sequel. Advances in physiology education.

