# Supplementary material for: Inflammatory cytokine-induced changes in neural network activity measured by waveform analysis of high-content calcium imaging in murine cortical neurons
Source: Sci Rep. 2017 Aug 22;7:9037. doi: 10.1038/s41598-017-09182-5 (PMC5567248; doi:10.1038/s41598-017-09182-5)
Supplement: Supplementary file 1 — Supplementary Materials [file 41598_2017_9182_MOESM1_ESM.pdf]

# Inflammatory cytokine-induced changes in neural network activity measured by waveform analysis of high-content calcium imaging in murine cortical neurons

Benjamin DS Clarkson[1], Robert J Kahoud[1,2], Christina B McCarthy[1], Charles L Howe*[,1,3,4,5]

## Supplementary Materials

## Supplementary Table S1.

| Gene Target | FORWARD | REVERSE | UPL PROBE |
|---|---|---|---|
| Glycine Receptor Glra1 (NM_001290821) | caacacaaggaactgcttcg | gatttagcatggggctcttg | 69 |
| Glycine Receptor Glra2 (NM_183427) | cataaggagttccttcgtctc | cgactttcacgagtaacatcttctt | 34 |
| Glycine Receptor Glra3 (NM_080438) | gggaagccgcactgttact | gagatcgcgcactgtttgt | 49 |
| Glycine Receptor Glrb (NM_010298) | ctgatgctagtgctgccaga | gtgcactctgaggccaaact | 105 |
| GABA A Receptor Gabra1 (NM_010250) | gcccactaaaattcggaagc | cttctgctacaaccactgaacg | 93 |
| GABA A Receptor alpha Gabra2 (NM_008066) | acaaaaagaggatgggcttg | tcatgacggagcctttctct | 103 |
| GABA A Receptor alpha Gabra3 (NM_008067) | cttgggaaggcaagaaggta | tggagctgctggtgttttct | 80 |
| GABA A Receptor alpha Gabra4 (NM_010251) | aaagcctcccccagaagtt | catgttcaaattggcatgtgt | 68 |
| GABA A Receptor alpha Gabra6 (NM_001099641) | caagtgtctttctggattaataaggag | gtcatggttaaaacagtggtgatt | 95 |
| GABA A Receptor beta Gabrb1 (NM_008069) | ccctctggatgagcaaaact | aattcgatgtcatccgtggta | 80 |
| GABA A Receptor beta Gabrb2 (NM_008070) | gggtctcctttggattaactatga | ggtcattgttaggacagttgtaattc | 84 |
| GABA A Receptor beta Gabrb3 (NM_008071) | ctccattgtagagcaccgtct | tcaatgaaagtcgaggataggc | 80 |
| GABA A Receptor gamma 2 Gabrg2 (NM_008073) | acagaaaatgacgctgtgga | catctgacttttggcttgtgaa | 68 |
| GABA A Receptor gamma 3 Gabrg3 (NM_008074) | atgcgacaccagcaagaac | caatggtgctgagtgtggtc | 9 |
| GABA A Receptor delta Gabrd (NM_008072) | cggagctgatgaacttcaaat | atgtagacgccccggttc | 11 |
| GABA A Receptor rho Gabrr1 (NM_008075) | tgcctgctagagtcccctta | ccgtgatgatggtggacat | 9 |
| GABA A Receptor pi Gabrp (NM_146017) | atttcaccctggtcaccgta | gctcaaattgcaaaaccaatc | 18 |
| GABA B Receptor 1 Gabbr1 (NM_019439) | gacattgatgtctccattctgc | gcagccctttgtaaccataga | 78 |
| GABA B Receptor 2 Gabbr2 (NM_001081141) | gaacatggcagcgaaagtct | ctggtacttgctgccaaaca | 91 |
| Glutamate Receptor AMPA 1 Gria1 (NM_008165) | agggatcgacatccagagag | tgcacatttcctgtcaaacc | 62 |
| Glutamate Receptor AMPA 2 Gria2 (NM_013540) | ggggaggtgattccaagg | cccccgacaaggatgtaga | 67 |
| Glutamate Receptor AMPA 3 Gria3 (NM_016886) | agccgtgtgatacgatgaaa | caaggtttacaggcgttcct | 31 |
| Glutamate Receptor AMPA 4 Gria4 (NM_019691) | ctgccaacagttttgctgtg | aaatggcaaacacccctcta | 48 |
| Glutamate Receptor Delta 1 Grid1 (NM_008166) | agacttttggcaaagacatgc | actgccattcaagcccttc | 95 |
| Glutamate Receptor Delta 2 Grid2 (NM_008167) | ccctaccgtgatgtcttttca | agaatgtccatgtcgccact | 1 |
| Glutamate Receptor Kainate 1 Grik1 (NM_146072) | tctggtttggcgttggag | tcctccaactattctggtcgat | 105 |
| Glutamate Receptor Kainate 2 Grik2 (NM_010349) | agtgccaccataccatccag | gctggcacttcagagacattc | 31 |
| Glutamate Receptor Kainate 3 Grik3 (NM_001081097) | cacttcatcttcaccactctgg | actcccgagtagcggtagg | 85 |
| Glutamate Receptor Kainate 4 Grik4 (NM_175481) | gccattgagtatggcacgat | tggtaacgggaattttggaa | 67 |
| Glutamate Receptor Kainate 5 Grik5 (NM_008168) | cccctcagctagcctcatct | gcctcgcaccagttcttcta | 40 |
| Glutamate Receptor NMDA 1 Grin1 (NM_008169) | catttagggctatcacctcca | cactgtgtctttttggttttgc | 78 |
| Glutamate Receptor NMDA 2A Grin2a (NM_008170) | attcaaccagaggggcgta | ttcaagacagctgcgtcatag | 48 |
| Glutamate Receptor NMDA 2B Grin2b (NM_008171) | gggttacaaccggtgccta | ctttgccgatggtgaaagat | 53 |
| Glutamate Receptor NMDA 2C Grin2c (NM_010350) | gaagcgggccatagacct | tggcagatccctgagagc | 94 |
| Glutamate Receptor NMDA 2D Grin2d (NM_008172) | tgcgatacaaccagccaag | agatgaaggcgtccagtttc | 25 |
| Glutamate Receptor NMDA 3A Grin3a (NM_001276355) | cacgaatcaaaaacaaatccaa | tgtgtttaatgctctgtgaaacc | 71 |
| Glutamate Receptor NMDA 3B Grin3b (NM_130455) | cgtcctacggagggaggt | ccagggacaccagcacat | 80 |
| Glutamate Receptor NMDA AP1 Grina (NM_023168) | ctcaaggaggctacccacag | tagttcccatgctgaggtga | 77 |

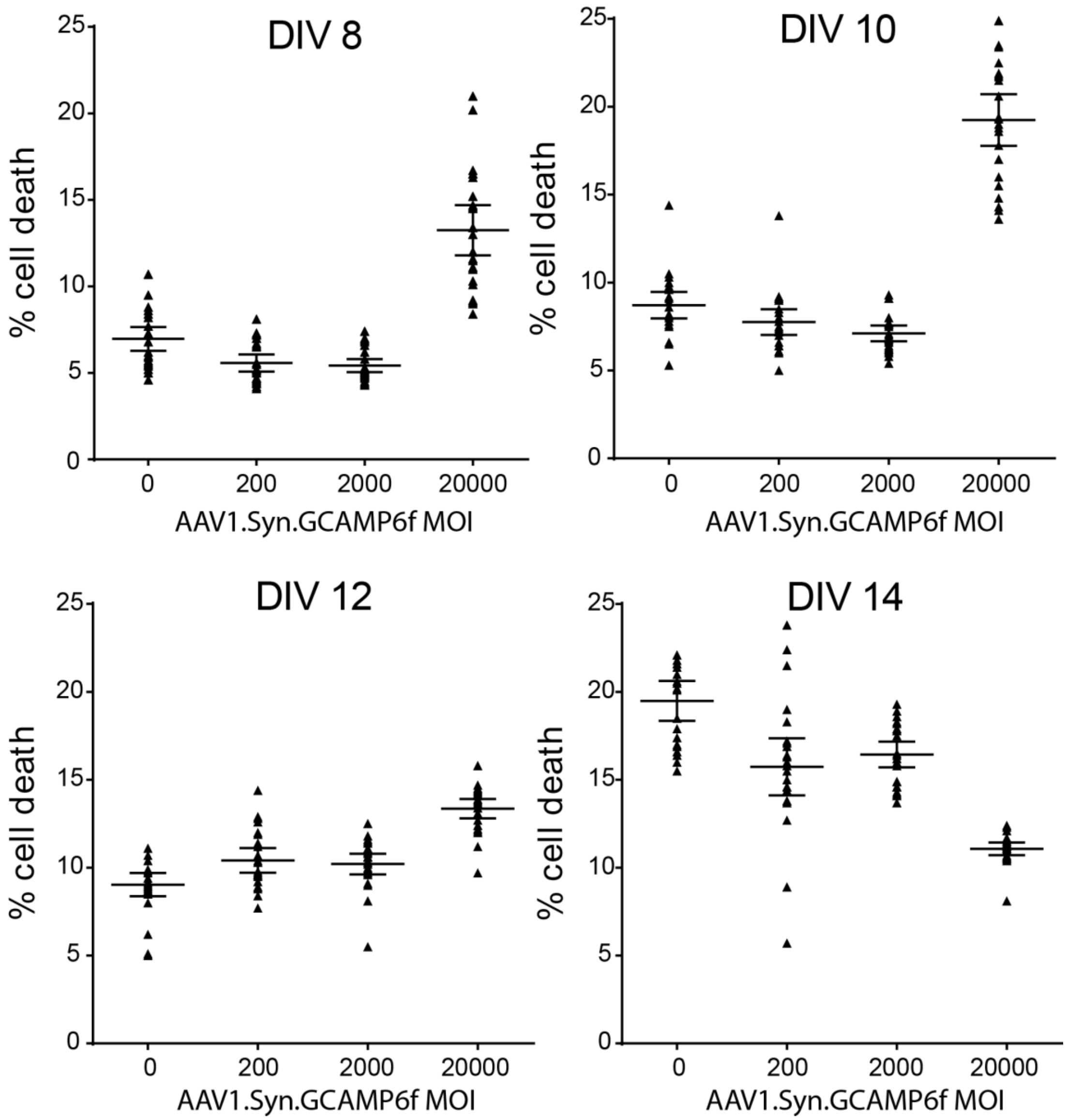


**Figures S1.**
Cell viability of cortical neuron cultures infected with AAV1.Syn.GCaMP6f at the indicated multiplicity of infection was assessed by measuring lactate dehydrogenase release into culture supernatant from DIV8-14 using a colorimetric assay. Data are normalized based on determining 100% LDH release following treatment of live cultures with 2% triton-x100. N = 24 per group. Error bars represent 95% confidence intervals.

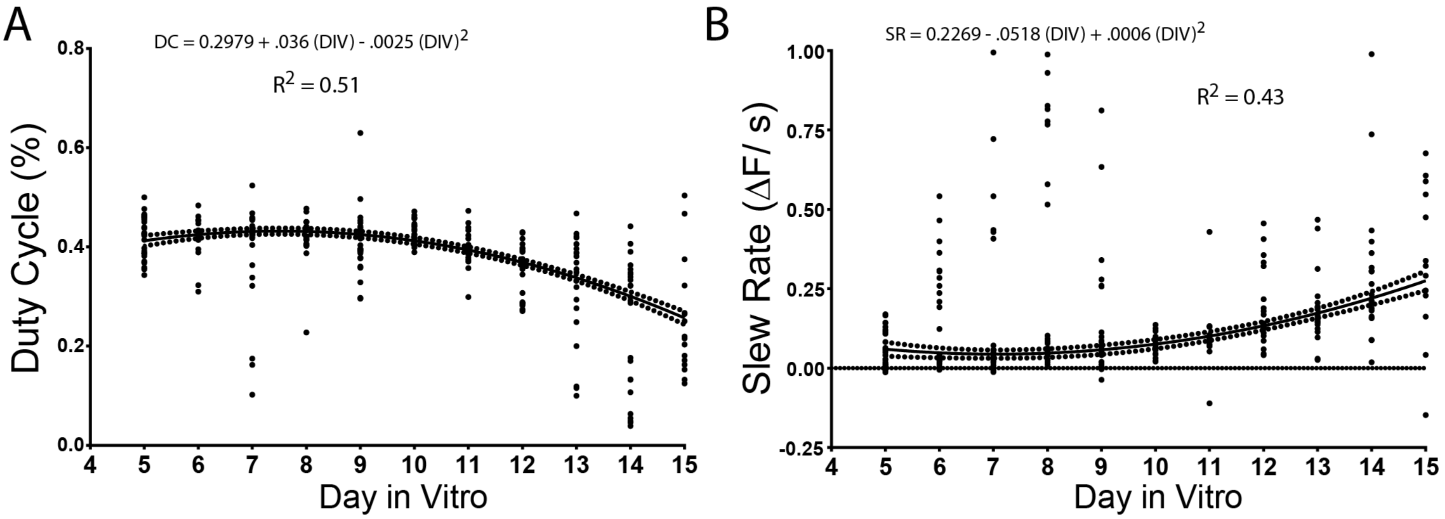


**Figures S2.**

Raw Δ fluorescence traces from neuronal cultures (DIV8-DIV15) were analyzed using the Matlab Signal Processing Toolbox, to extract duty cycle (A) and slew rate (B). Plots show averaged values from > 16 acquisitions at each time point taken from 4 independent experiments. Solid lines represent quadratic regressions with 95% CI (dotted line). Best fit equations are shown along with $R^2$ values .

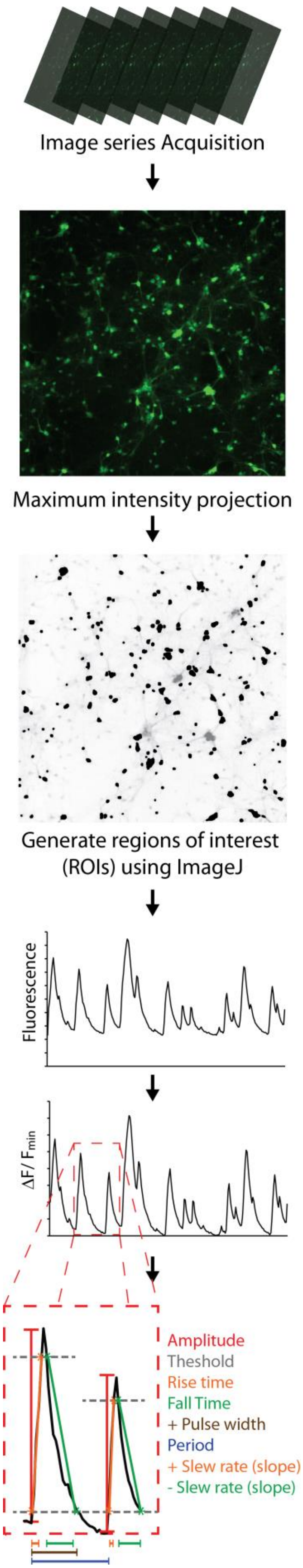


**Figure S3.**

An overview of imaging and analysis workflow is shown. Briefly, maximum intensity projections of image series acquisitions are exported from Zen software, batch processed in ImageJ to threshold and segment images into regions of interest. An example of mean fluorescence trace for one ROI over the acquisition is shown before and after normalization to minimum fluorescence. Graphical depiction of extracted waveform parameters is shown below.

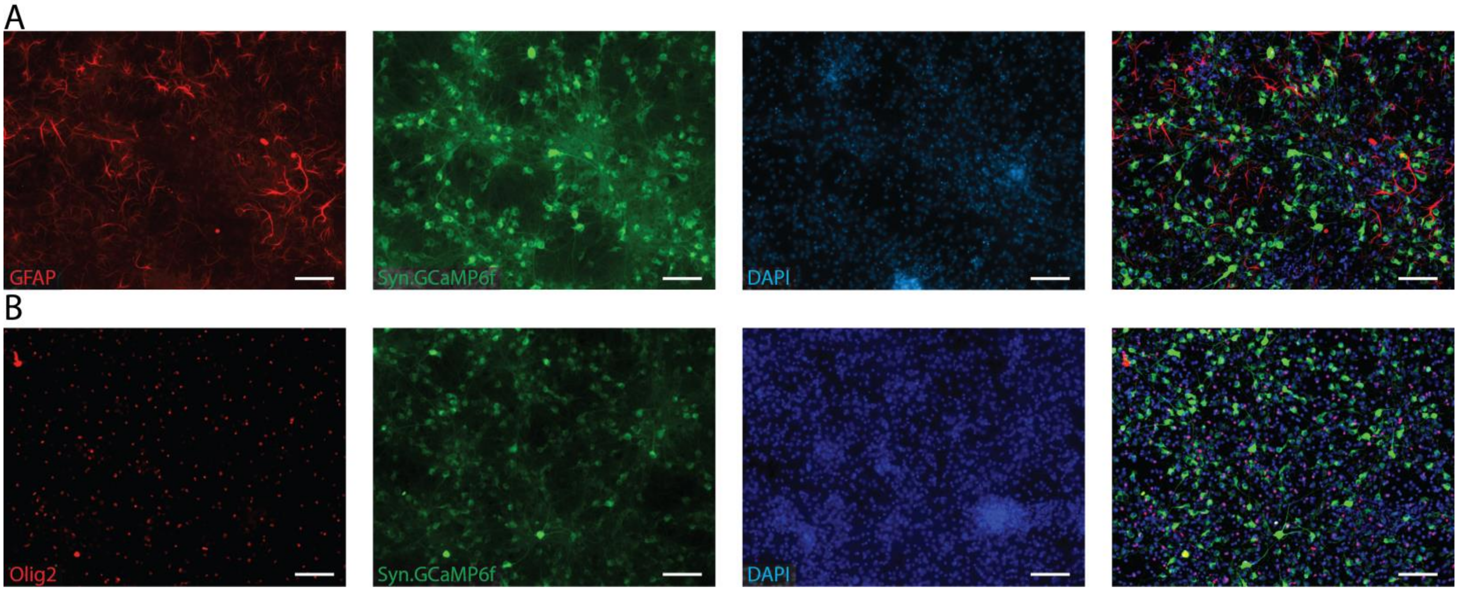


**Figure S4.**

DIV 15 cortical neuron cultures were fixed with 4% PFA and permeabilized with 0.1% triton-x100. Cells were then immunostained for GFAP (astrocytes, A) or Olig2 (oligodendroglia and progenitor cells, B) and counterstained with DAPI. Representative images shown were acquired on an Axio Observer epifluorescent microscope. Scale bars 100 microns.

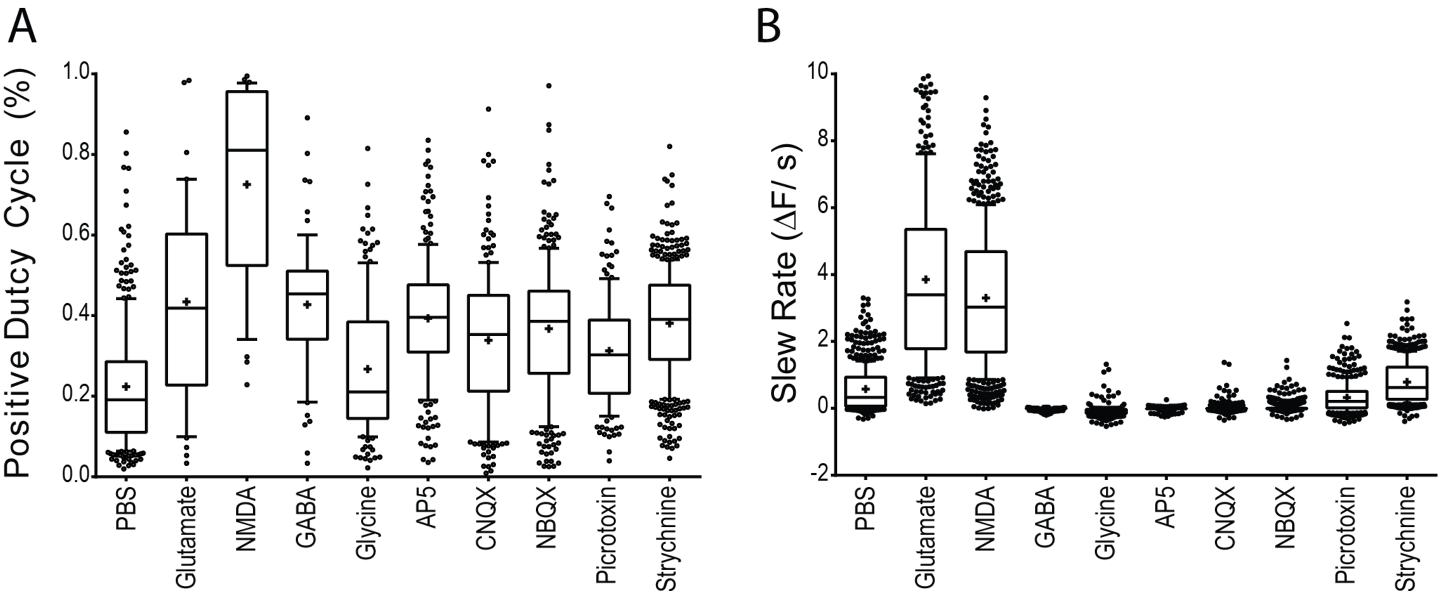


**Figure S5.**
DIV11 cortical neurons were left untreated or acutely treated with PBS, glutamate (23 μM), NMDA (35 μM), AP5 (29 μM), CNQX (3 μM), GABA (27 μM), glycine (1.7 mM), picrotoxin (2.4 μM), or strychnine (6.3 μM). Average positive duty cycle (% of total period), and slew rate are plotted. Box plots show median (line), average (+), and interquartile range; error bars represent 10-90% range with outliers shown. Data are representative of 3-6 treatment replicates across two independent experiments.

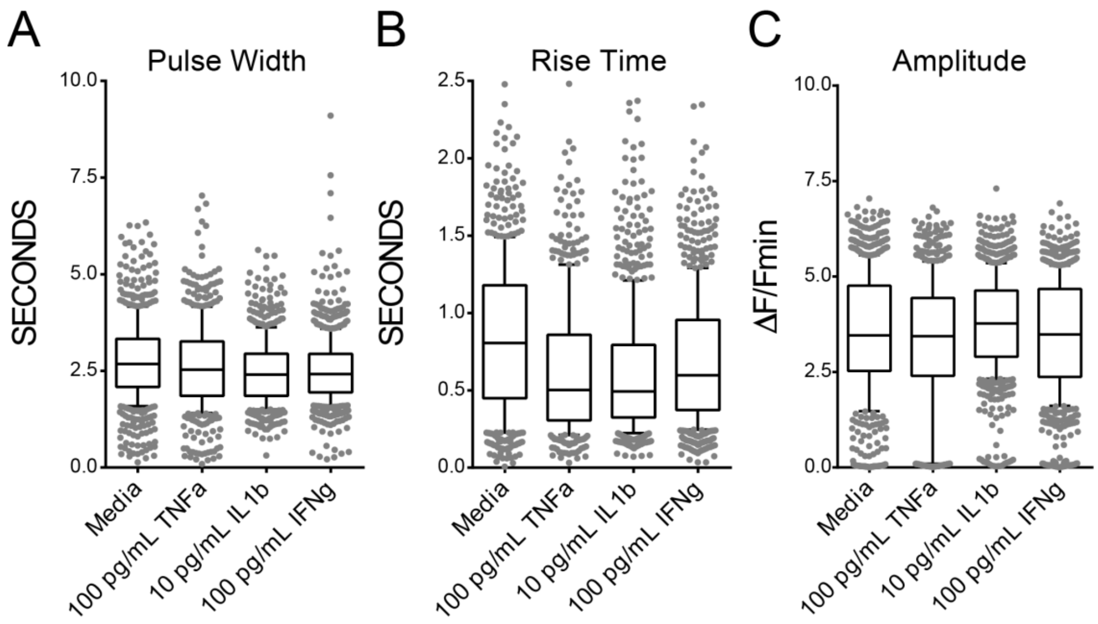


**Figure S6.**

Following DIV0 infection with 2000 MOI AAV1.Syn.GCaMP6f, DIV 12 cortical neurons were treated with media or the indicated concentration of TNFα, IL1β, or IFNγ for 24 hours prior to acquisition as in Figure 5. Average pulse width (A), rise time (B) and amplitude (C) are plotted. Box plots show median (line and interquartile range; error bars represent 10-90% range with outliers shown.
